# Integrin-mediated adhesive properties of neutrophils are reduced by hyperbaric oxygen therapy in patients with chronic non-healing wound

**DOI:** 10.1371/journal.pone.0237746

**Published:** 2020-08-18

**Authors:** Monica Baiula, Roberto Greco, Lucia Ferrazzano, Alberto Caligiana, Klarida Hoxha, Daniele Bandini, Pasquale Longobardi, Santi Spampinato, Alessandra Tolomelli

**Affiliations:** 1 Department of Pharmacy and Biotechnology, Alma Mater Studiorum - University of Bologna, Bologna, Italy; 2 Department of Chemistry “Giacomo Ciamician”, Alma Mater Studiorum -University of Bologna, Bologna, Italy; 3 Hyperbaric Centre, Ravenna, Italy; University of Arkansas for Medical Sciences College of Pharmacy, UNITED STATES

## Abstract

In recent years, several studies suggested that the ability of hyperbaric oxygen therapy (HBOT) to promote healing in patients with diabetic ulcers and chronic wounds is due to the reduction of inflammatory cytokines and to a significant decrease in neutrophils recruitment to the damaged area. α_4_ and β_2_ integrins are receptors mediating the neutrophil adhesion to the endothelium and the comprehension of the effects of hyperbaric oxygenation on their expression and functions in neutrophils could be of great importance for the design of novel therapeutic protocols focused on anti-inflammatory agents. In this study, the α_4_ and β_2_ integrins’ expression and functions have been evaluated in human primary neutrophils obtained from patients with chronic non-healing wounds and undergoing a prolonged HBOT (150 kPa per 90 minutes). The effect of a peptidomimetic α_4_β_1_ integrin antagonist has been also analyzed under these conditions. A statistically significant decrease (68%) in β_2_ integrin expression on neutrophils was observed during the treatment with HBO and maintained one month after the last treatment, while α_4_ integrin levels remained unchanged. However, cell adhesion function of both neutrophilic integrins α_4_β_1_ and β_2_ was significantly reduced 70 and 67%, respectively), but α_4_β_1_ integrin was still sensitive to antagonist inhibition in the presence of fibronectin, suggesting that a combined therapy between HBOT and integrin antagonists could have greater antinflammatory efficacy.

## Introduction

Hyperbaric oxygen (HBO) therapy has emerged in the last years as an innovative approach and an effective adjunctive therapy for the treatment of different pathologies. The oxygen pressure applied in the chamber is usually from 165 to 275 kiloPascal (kPa– 1.7 to 2.8 absolute atmospheres, ATA) and the first effect of pressurizing the human body is the increase of partial pressure of gases and the decrease of volume of gas-filled spaces according to Boyle’s law [1, 2].

The additionally available oxygen has the ability to restore oxygenation in areas where hypoxia or hypoperfusion occur, and it can help damaged tissue to heal [1]. Moreover, increased oxygen levels, that lead to changes in reactive oxygen species (ROS) and nitrogen species (RNS) production during HBO therapy (HBOT), are essential to stimulate specific repair functions of macrophages, neutrophils and fibroblasts in the healing process [3–7]. In addition, HBOT regulates the inflammatory response (reduction of NLRP3 inflammasome, proinflammatory cytokines, including IL-1β, IL-6 e IL-18, TNF*α*) [8–10].

HBOT has been successfully employed to control non-healing diabetic ulcers and chronic wounds, significantly minimizing the number of amputations relative to standard wound care alone in diabetic population [2].

Wound healing is a complex process that involves growth factors, components of the extracellular matrix and several cell types. Inflammatory cytokines, such as tumor necrosis factor-α (TNF-α) and interleukin-1β (IL-1β) are often present at high levels in the site of inflammation, like in chronic wounds [11]. The immune system is involved in all the steps of tissue repair [12] Inflammatory response evolves in the leukocyte-adhesion cascade, primarily mediated by two major adhesion receptor families, selectins and integrins [13].

Neutrophils play a crucial role in wound healing process by sensing their environment and responding to the extracellular signals by adhesion, migration and other effector functions [12]. After ending their role at the site of inflammation, neutrophils undergo apoptosis and are removed by macrophages; this latter event is considered a strong signal for inflammation resolution. Although fighting infection, neutrophils can also have harmful effects inducing damage in the inflamed tissue and leading to a delay in healing process [12] and chronic inflammation.

Neutrophils express integrins on their surface, significantly contributing to the recruitment phase. Among them, β_2_ integrin family members, including α_L_β_2_ and α_M_β_2_, bind to endothelial intercellular adhesion molecule-1 (ICAM-1) and α_4_β_1_ integrin recognizes vascular cell adhesion molecule-1 (VCAM-1) expressed on endothelial cells [14]. Moreover, α_4_β_1_ integrin and β_2_ integrins are involved in the onset and resolution of inflammatory process mediating the adhesion of monocytes, lymphocytes and neutrophils to the blood vessels [15].

Several studies carried out on animal models, have evidenced that neutrophil recruitment is significantly reduced by treatment with HBO in case of damage induced by ischemia and reperfusion [16–19]; in other studies HBOT induces the reduction of tissue necrosis [20, 21] and lipid peroxidation [22].

Moreover, it has been shown that HBO treatment inhibits ischemia reperfusion-induced neutrophil adhesion to endothelium by blocking β_2_ integrin polarization [23, 24] and may also reduce leukocytes recruitment as it impaired adhesion molecule function by S-nitrosation [25]. Conversely, the role played by α_4_β_1_ integrin in neutrophil-mediated adhesion to endothelium has been poorly investigated but an important role of α_4_β_1_ in β_2_ integrin-independent migration of neutrophils across heart endothelium has been demonstrated *in vitro*, suggesting a similar *in vivo* situation in neutrophil trafficking in reperfused myocardium [26].

HBOT represents an effective therapy for chronic wounds as it reduces inflammation and accelerates healing [27], probably involving integrins. In the present study, we investigated whether HBOT could exert its effects by modulating functions of α_4_β_1_ and β_2_ integrins expressed on neutrophils obtained from patients with chronic non-healing ulcers. In a previous study, Thom et al. [28] isolated polymorphonuclear leukocytes (PMN) from young healthy volunteers exposed to only one session of HBO. They observed a reduction in cell adhesion mediated by β_2_ integrins without any variation of its expression. Our aim is to evaluate the role of integrin-mediated adhesion by characterizing the expression of integrin receptors on neutrophils during the HBOT and by analyzing the effect of a peptidomimetic α_4_β_1_ integrin antagonist under these conditions. If integrins are a target for both HBOT and synthetic antagonist that blockade their activation, a joint therapy could be hypothesized, leading to a faster and stronger decrease of inflammation. To this purpose, expression and function of α_4_β_1_ and β_2_ integrins were investigated for the first time in human primary neutrophils isolated from the blood of patients with chronic non-healing ulcer undergoing HBOT or standard wound therapy alone. Expression of these integrins was monitored in patients, before the beginning of HBOT exposure and during the therapy using specific antibodies towards α_4_ integrin or β_2_ integrin family. Patient wound area size was measured and pro-inflammatory cytokine levels were evaluated both in neutrophils and in plasma. Furthermore, *in vitro* cell adhesion assays were performed in the presence of a peptidomimetic integrin antagonist previously developed by our group [29], to investigate if the HBO treatment may influence neutrophil recruitment and adhesion mediated by α_4_β_1_ integrin.

## Materials and methods

### Patients’ recruitment

The study protocol was conformed to the ethical guidelines of the 1975 Declaration of Helsinki.

The patients followed exclusively the therapy prescribed by their medical doctor, following the indications of the Italian public health system. No additional treatments were done for the purposes of the study which did not change any therapeutic protocol nor interfere with the healing progress. The patients of the HBOT group were submitted to the prescribed HBOT and to blood sampling following the standard protocols approved by the Institutional Ethics Review Board of the "Comitato Etico della Romagna" (C.E.ROM.) (Version 1.3 of June 13, 2018 approved by C.E.ROM. Ethical Committee, Reg. Sperimentazioni 2125 Prot. N.4533/2018/I.5/150), which has full access to the data in order to check conformity of the study to the current regulation.

Thirty patients from Hyperbaric Centre (Ravenna, Italy) were enrolled in the study between July 2018 and January 2019 in two different groups: control group patients (n = 15) received standard wound care alone (as prescribed by their medical doctor), whereas HBOT group patients (n = 15) received HBOT in addition to conventional wound treatment. The inclusion criteria were: adults 18 years or older and chronic wounds that fail to demonstrate improvement (>50% wound area reduction) after a minimum of 4 weeks of standard wound therapy. The exclusion criteria were: symptoms of bacterial infection, malignancy, pregnancy, medications that can adversely affect healing including anticonvulsants, steroids, antibiotics, angiogenesis inhibitors and NSAIDs, such as drugs known to promote healing including vitamins, thyroid hormone and iron. Contraindication for HBOT were: claustrophobia, uncontrolled diabetes, middle ear problems, glaucoma, heart failure, pacemaker, untreated pneumothorax, chronic obstructive pulmonary disease, pulmonary emphysema with retention of CO_2_, prothesis and seizures. Patients in the control group were matched by gender, age and ethnicity. Exclusion criteria for control group patients were the same for patients in HBOT group.

The patients allowed donating blood samples and were informed of the aim of the study and completed the written informed consent process before enrolling in the study. At the end of the study, all individuals received personal information about the results relative to their own samples.

The following demographic information was collected for all recruited patients at baseline: gender, age, smoker/nonsmoker, and other medical conditions. Photographs of the ulcer were taken at various times. After sharp wound debridement with a sterile scalpel, the wound’s surface area was calculated by VISITRAK^™^ Digital System (Smith & Nephew). For each patient, the ulcer was graded and staged by a clinician, as described below.

### Sample size

Sample size was based on a power analysis using G*Power [30]. The power was set at 0.8 to limit the risk of committing a type II error to 20%, the α level was set at 0.05. The effect size was set at 0.37, and the number of samples for each group was calculated to be 15 to detect statistically significant differences (P value of 0.05) between 2 groups. In this study, 30 patients were selected and allocated to 2 study groups (control and HBOT group).

### Hyperbaric oxygen therapy protocol

HBOT was conducted at the Hyperbaric Centre in Ravenna, Italy. The protocol procedure consisted of 15 HBO exposures in a multiplace hyperbaric chamber (daily session, five per week from Monday to Friday). HBOT group patients breathed 100% oxygen at 245 kPa per 90 minutes in cycles for 20 minutes separated by 3 minutes medical air breathing intervals.

### Classification of wounds

In this study ulcers were graded using Falanga Wound Bed Preparation Score (Staging copyrighted [31]) that provides staging of varying degrees based upon descriptions and characteristics of ulcers (Table 1) [31]. Staging of the wounds was done by combining the score of the wound bed appearance with that of the wound exudate (Table 1). Clinical assessment of wound conditions was conducted for HBOT group before HBO treatment (T_0_), immediately after the fifteenth HBOT session (T_15_) and one month after ending HBOT (T_1M_); for control group during the first evaluation by a medical doctor (T_0_) and after fifteen days of conventional wound therapy (T_15_).

**Table 1:** Cutaneous ulcers were graded using Falanga wound classification system.

		Wound Bed Characteristics		
Wound appearance		Granulation tissue	Fibrinous tissue	Eschar
A		100%	–	–
B		50–100%	+	–
C		< 50%	+	–
D		Any amount	+	+
Wound Exudate Score	Extent of Control	Exudate Amount	Dressing requirement	
1	Fully	None/minimal	No absorptive dressing required. If clinically feasible, dressings could stay on for up to a week	
2	Partially	Moderate amount	Dressing changes required every 2–3 days	
3	Uncontrolled	Very exudative wound	Absorptive dressing changes required at least daily	

Adapted from [31].

### Neutrophil isolation from peripheral blood

Venous blood samples were obtained from the antecubital vein of participants in EDTA containing vacutainers. Neutrophil isolation and further experiments were performed at the Department of Pharmacy and Biotechnology, University of Bologna (Bologna, Italy). Blood samples were obtained for patients in control group during the first evaluation by a clinician (T_0_) and after fifteen days of conventional wound therapy (T_15_), while for HBOT group patients, before (T_0_) and immediately after the fourth (T_4_), the eighth (T_8_), the twelfth (T_12_) and the fifteenth (T_15_) HBOT sessions. In addition, one month after ending HBOT (T_1M_) another blood sample was collected from HBOT group patients. For neutrophil isolation, blood was carefully layered on top of an equal volume of Lympholyte^®^-poly (Cedarlane) and centrifuged at 500 g for 35 min at 20–25°C, as previously described [32]. Plasma was carefully removed and store at -80°C for further analysis. Neutrophils were transferred in a clean tube and were resuspended in 10 mL of HBSS (Hanks’ Balanced Salt Solution, Life Technologies Italia) without Ca^2+^/Mg^2+^ and centrifuged at 350 g for 10 minutes. To lyse the residual red blood cells (RBCs), 2 mL Red Cell Lysis Buffer (Roche) were added and the cells were resuspended vortexing at low speed to avoid neutrophils activation. Neutrophils were centrifuged at 250 g for 5 min, resuspended in HBSS without Ca^2+^/Mg^2+^ and adjusted to desired concentration.

Cell viability was determined using Annexin V/7-AAD assay (Guava Nexin Reagent, Millipore) as previously described [33] and was >98%. Differential analysis of cells retrieved using this procedure showed >98% granulocytes of which >95% were neutrophils. Neutrophils were stored at room temperature and used for functional tests (cell adhesion assay and flow cytometry analysis) within 4 h of collection. An aliquot of the purified neutrophils was immediately stored at -80°C and used for mRNA extraction.

### Neutrophil adhesion assay

Adhesion assays on purified neutrophils were performed as previously described [29, 33]. Briefly, black 96-well plates were coated overnight at 4°C with fibronectin (FN) or fibrinogen (Fg) (both 10 μg/mL) to study respectively adhesion mediated by α_4_β_1_ and β_2_ integrins. Neutrophils were counted and stained with CellTracker green CMFDA (12.5 μM, 30 min at 37 °C, Life Technologies Italia). Thereafter, cells were plated (50000/well) on coated wells and incubated for 30 min at 37 °C. After three washes, adhered cells were lysed with 0.5% Triton X-100 in 1% BSA (bovine serum albumin) in HBSS (30 min at 4 °C) and fluorescence was measured (Ex485 nm/Em535 nm). To evaluate the ability of ligand 1 [29] (named here RG66) to inhibit neutrophils’ adhesion, cells were pre-incubated with various concentrations (10^−4^–10^−10^ M) of RG66 or vehicle (methanol) for 30 min at 37°C before plating cells into coated wells. Neutrophil adhesion assays were also carried out in the presence of an anti-human β_2_ or α_4_ integrin antibody (both 5 μg/mL; purified mouse anti-human CD18 and purified mouse anti-human CD49d antibody, BD Pharmingen). Experiments were carried out in triplicate. Data analysis and IC_50_ values were calculated using GraphPad Prism 5.0 (GraphPad Software, San Diego, CA, USA).

### Flow cytometry analysis

Purified neutrophils were suspended in 1% BSA in HBSS at the concentration of 10^6^ cells/mL (100 μL/sample) and incubated with FITC-labeled anti-α_4_ integrin antibody (5 μL/sample, FITC Mouse anti-human CD49d, BD Pharmingen) or FITC-labeled anti-β_2_ integrin antibody (15 μL/sample, FITC Mouse anti-human CD18, BD Pharmingen) for 45 min at 4°C, as previously described [33]. After two washes with 1% BSA in HBSS, cells were resuspended in PBS and analyzed in a Guava EasyCyte Flow Cytometer (Millipore) and 10000 cells/sample were analyzed. Data were normalized with the relative fluorescence for nonspecific binding evaluated by exposing the cells to an isotype control monoclonal antibody FITC mouse IgG (Becton Dickinson Italia) and set to 0.

In another set of experiments purified neutrophils were suspended in 1% BSA in HBSS at the concentration of 10^6^ cells/mL (100 μL/sample) and incubated with the conformational sensitive phycoerythrin (PE)-labeled HUTS-21 monoclonal antibody (20 μL/sample, PE mouse antihuman CD29 antibody, BD Pharmingen) for 45 min at room temperature. Neutrophils were washed twice with 1% BSA in HBSS, resuspended in PBS and analyzed at the flow cytometry. 10000 cells/sample were analyzed. Data were normalized to nonspecific binding relative fluorescence evaluated by exposing the cells to an isotype control mAb (monoclonal antibody) and set to 0.

### Quantitative real time PCR

Total RNA was extracted from purified neutrophils with TRI Reagent (Sigma-Aldrich) and quantified using a NanoDrop spectrophotometer (ThermoFisher Scientific). For each sample, 1–2 μg of total RNA was treated with RNase-free DNase as previously described [34]. The RNA samples were then converted into cDNA using High-Capacity cDNA Reverse Transcription Kits (Life Technologies Italia), according to the manufacturer’s instructions. Real-time PCR was performed using GoTaq^®^ qPCR Master Mix (Promega Corporation, Madison, WI, USA). The protocol consisted of: (i) for L19 and TNF-α: denaturation at 95°C for 10 minutes, followed by 40 cycles of 95°C denaturation (15 seconds) and 60°C annealing/extension (1 minute); (ii) for α_4_ integrin: denaturation at 95°C for 10 minutes, followed by 40 cycles of 95°C denaturation (15 seconds), 67°C annealing (20 seconds) and 68°C (20 seconds); (iii) for IL-1β: denaturation at 95°C for 10 minutes, followed by 40 cycles of 95°C denaturation (30 seconds), 68°C annealing (30 seconds) and 72°C (30 seconds). No-template controls and DNA melting curve analysis were used as controls to ensure the lack of contaminating DNA in the RNA preparations and to rule out primer-dimer formation, respectively. To amplify integrin and cytokine targets the following primers were used: α_4_ integrin: sense primer (5′-GTCGCATCCCGTGCAACTTTG-3′) and antisense primer (5′-GCTGTGCAGCACGACCGAGT-3′), amplifying a 243 bp fragment; TNF-α: sense primer (5′-CTTCTCCTTCCTGATCGTGG-3′) and antisense primer (5′-TCTCAGCTCCACGCCATT-3′), amplifying a 255 bp fragment [35]; IL-1β: sense primer (5′-CAAGGGCTTCAGGCAGGCCG-3′) and antisense primer (5′-TGAGTCCCGGAGCGTGCAGT-3′), amplifying a 213 bp fragment [36]. To amplify β_2_ integrin cDNA, primer sequences were from PrimerBank [37]; a sense primer (5′-TGCGTCCTCTCTCAGGAGTG-3′) and an antisense primer (5′-GGTCCATGATGTCGTCAGCC-3′) amplifying a 187 bp fragment were used.

As reference control, a 169 bp fragment of the L19 ribosomal protein was amplified using a sense primer (5′-CTAGTGTCCTCCGCTGTGG-3′) and an antisense primer (5′-AAGGTGTTTTTCCGGCATC-3′) [38]. For data analysis, relative expression of RT-PCR products was determined using the ΔΔC_T_ method [39], as previously described [40]; the threshold cycle (Ct) values were normalized both on the basis of L19 content and on the values derived from T_0_ sample. Each sample was tested in triplicate. Primers were synthesized by Sigma-Aldrich.

### Cytokine quantification in plasma by ELISA

Cytokine protein levels (TNF-α and IL-1β) were determined in plasma of patients using ELISA kits (Invitrogen, LifeTechnologies Italia, Monza, Italy) according to the manufacturer’s instructions. Briefly, 50μL/well of sample were added to a 96-well plate together with 100 μL of biotin conjugated primary antibody; the plate was then incubated for 2 hours at room temperature. After washing four times, 100 μL of streptavidin-HRP solution were added and the plate was incubated for 30 min at room temperature. The wells were washed 4 times and afterwards 100 μL of stabilized chromogen were added and incubated for 25 min at room temperature. After the addition of 100 μL of stop solution, absorbance was read at 450 nm using an EnSpire Multimode Plate Reader (PerkinElmer, Waltham, MA, USA). The calculated overall intra-assay coefficient of variation was 4.4% for TNF-α and 4.4% for IL-1β; the inter-assay coefficient of variation was calculated to be 7.5% for TNF-α and 6.7% for IL-1β.

### Synthesis and bioactivity of α_4_β_1_ synthetic ligand

The synthesis of *(R)-*RG66 has been previously reported [29] via a multistep synthesis starting from an enantiopure (*3R*,*Z*)-tert-butyl 3-(allylamino)-2-ethylidene-4-methylpentanoate. The detailed description of the synthetic protocol has been reported in S1 Fig. The biological evaluation showed a strong dependence of the bioactivity on the ring stereochemistry could be detected, since (*S*)-**1** turned out to be completely inactive [29].

### Data and statistical analysis

All assays were carried out in triplicate for individual sample at each time point/person and n refers to the number of individuals. Continuous variables are presented as mean ± standard deviation when normally distributed; data were tested using one-way ANOVA followed by Newman-Keuls post-test or using standard Student t test. In addition, data are presented as median and range and analyzed using Mann-Whitney’s test when non normally distributed. Categorical data were analyzed using χ^2^-square test. Data analysis and IC_50_ values referring to adhesion assays in the presence of RG66 compound were fitted using sigmoidal dose-response equation using GraphPad Prism software. Statistical analyses were performed using GraphPad Prism (version 5.0; GraphPad Software, Inc., La Jolla, CA, USA). P < 0.05 was considered significant.

## Results

### Demographic and clinical data

30 patients, 13 men and 17 women, (age 74.5 ± 12.7 years) presenting a chronic non-healing wound condition were recruited and volunteered to participate in the study. All the patients completed the study and no patient was excluded from the data analysis. Demographic parameters such as age, gender and clinical data are summarized in Table 2.

**Table 2:** Demographic and clinical data of patients enrolled in the study.

Demographic data	Control group	HBOT group	P
Number of subjects	15	15	1
Age (years)^a^	77 (50–88)	76 (60–91)	0.98
Female/Male^b^	8/7	9/6	0.71
Smokers	3	4 (ex)	0.78
**Comorbidities**^b^			
Diabetes	type-1 0	type-1 2	
	type-2 2	type-2 3	
Hypertension	6	6	
Obesity	3	3	
Venous insufficiency	4	3	
Glaucoma	1	3	
Cardiomyopathy	4	4	
Hypothyroidism	2	4	
HBV/HCV positive	0/1	2/0	
Rheumatoid arthritis	0	1	

^a^Age is expressed as median (range).

^b^ Gender and comorbidities are expressed as number of individuals.

Age distributions, demographic and clinical characteristics of patients were compatible between HBOT and control groups (P > 0.05).

Overall, the wounds of the patients enrolled in the study were caused by different etiologies: 23% caused by diabetes, 23% by venous insufficiency, 15% by critical limb ischemia, 24% by trauma, and 15% by vasculitis.

As regards comorbidities, seven of the subjects suffered from diabetes (2 of type-1 and 5 of type-2), twelve of them of hypertension, six of obesity, seven of venous insufficiency, eight of cardiomyopathy and six of hypothyroidism not requiring thyroid hormones. Three subjects were smokers and four previous smokers. Common sites of wound were leg (10 patients), foot (7 patients), great toe (7 patients), and other sites (6 patients). Before beginning the HBOT, all participants passed a standard medical and physical revision at the Hyperbaric Centre in Ravenna. All the patients, both in control and HBOT groups, received standard wound care, i.e. wounds were cleaned gently, minimizing chemical or mechanical trauma, at low pressure (4–15 psi) with saline solution and daily sterile sharp debridement with scalpel, curette or scissors was performed for the necrotic tissue removal, with caution to avoid excess tissue damage which may delay healing, to get a well-bleeding granulating base.

### Integrin expression on neutrophils deriving from patients undergoing HBOT

In order to study the effect of HBOT on neutrophil integrin expression, these cells were isolated from blood samples deriving from patients with a chronic non-healing wound, receiving standard wound care alone (control group) or undergoing HBOT (HBOT group). For control group patients, blood samples were collected during the first wound evaluation (T_0_) and after fifteen days of conventional wound therapy (T_15_); for patients in the HBOT group, blood samples were obtained before (T_0_) and immediately after the fourth (T_4_), the eighth (T_8_), the twelfth (T_12_) and the fifteenth (T_15_) HBOT treatment (at the end of three weeks; five exposures/week); moreover, the last blood sample was drawn one month after ending HBOT (T_1M_).

**Fig 1:**
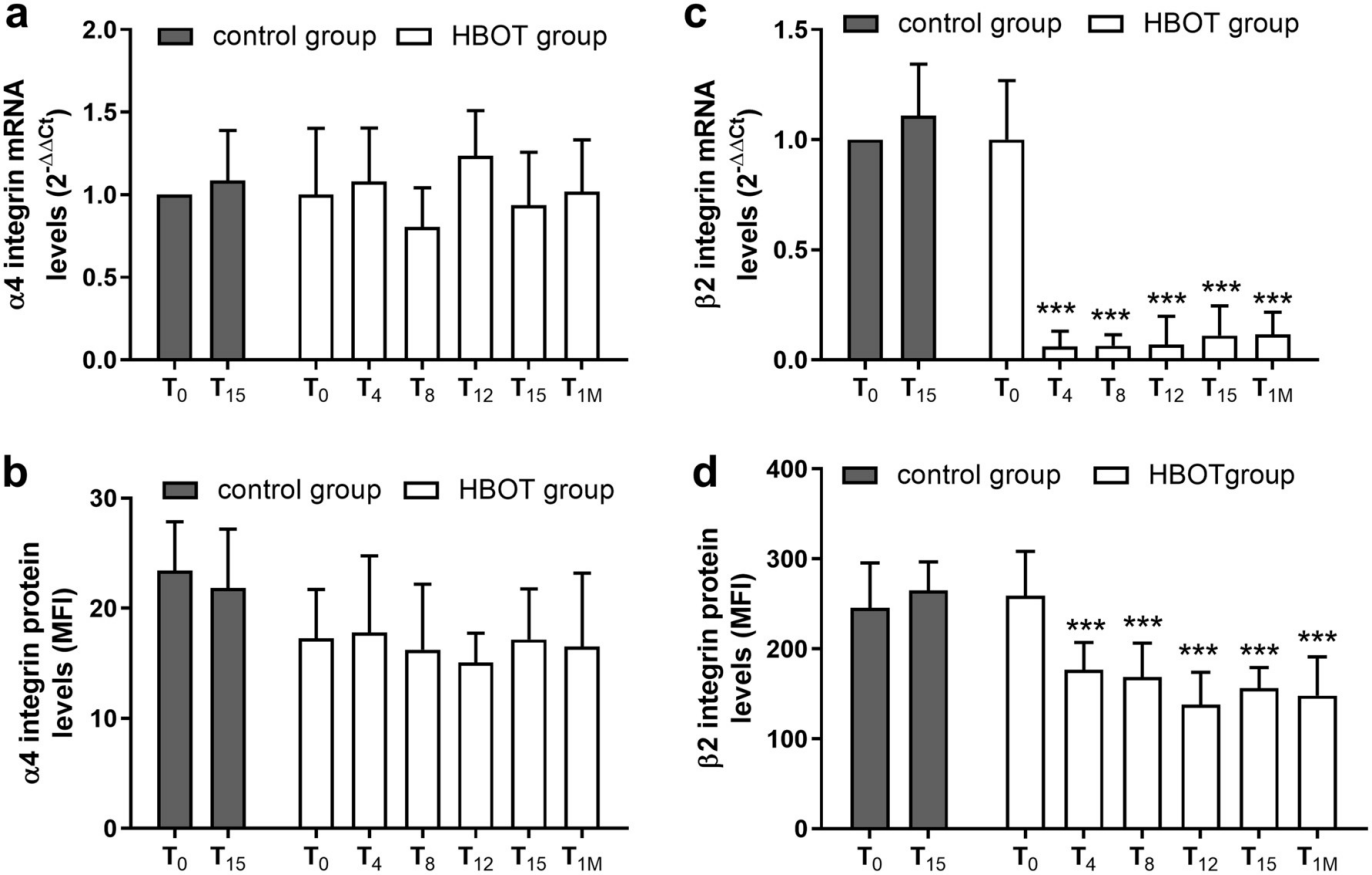
HBOT does not modify α_4_ integrin levels (panels a and b) but reduces β_2_ expression (panels c and d) in neutrophils deriving from patients receiving standard wound care alone (control group) and patients undergoing HBOT for 15 sessions (HBOT group). The decrement of β_2_ integrins was maintained up to one month after ending HBOT. The effects of HBOT on integrin expression were evaluated both by qPCR (mRNA levels; panel a, c) and by flow cytometry (measuring integrin expressed on cell surface; panel b, d). Results from qPCR are expressed as mean ± standard deviation of individual samples, carried out in triplicate at each time point (control group n = 15; HBOT group n = 15). Data from flow cytometry analysis are expressed as mean fluorescence intensity (MFI) ± standard deviation of individual samples carried out in triplicate at each time point (control group n = 15; HBOT group n = 15). MFI values for respective isotype control monoclonal antibody were set to 0. *** p < 0.001 versus T_0_, both control and HBOT group.

As shown in Fig 1, HBOT did not alter α_4_ integrin expression in primary human neutrophils, both at the mRNA and protein levels (Fig 1, panels a and b) nor induced any significant variation in α_4_ integrin expression, throughout the duration of HBO treatment. No significant change in α_4_ integrin expressed on neutrophils was observed in both control and HBOT group patients.

On the contrary, β_2_ integrins were significantly reduced by HBOT both at mRNA and protein levels; this decrement was maintained up to the end of HBO treatment and also one month after the last HBOT session (Fig 1, panels c and d). Neutrophils deriving from patients receiving conventional wound care alone (control group) did not show any changes in β_2_ integrins expression.

### Effects of HBOT on integrin-mediated adhesive properties of neutrophils

**Fig 2:**
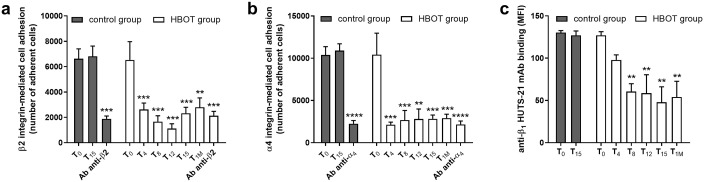
HBOT reduces significantly integrin-mediated neutrophil adhesion to fibrinogen (Fg) or fibronectin (FN). Integrin-mediated adhesion was decreased in neutrophils obtained from patients belonging to HBOT group; no changes were observed in control group patients. The effects of HBOT on neutrophil adhesion mediated by β_2_ (panel a) or α_4_β_1_ (panel b) integrins were evaluated by adhesion assay to Fg or FN respectively, as described in method section. Adhesion mediated by β_2_ or α_4_β_1_ integrin is significantly prevented in neutrophils treated with a monoclonal antibody anti-β_2_ or anti-α_4_, respectively. HBOT exposure significantly reduced anti-β_1_ HUTS-21 mAb binding to neutrophils, namely changing α_4_β_1_ integrin conformation (panel c). Mean fluorescence intensity (MFI) due to the anti-β1 integrin mAb PE conjugated HUTS-21 binding in the presence of fibronectin (10 μg/mL) was measured. Non-specific binding of an isotype control PE conjugated mAb added to neutrophils produced an MFI of 12 ± 3 that was subtracted from all samples. Data are expressed as mean ± standard deviation of individual samples, carried out in triplicate at each time point (control group: n = 15; HBOT group n = 15). ** p < 0.01; *** p < 0.001; **** p < 0.0001 versus T_0_, both control and HBOT group.

During an inflammatory process, neutrophils adhere and transmigrate through blood-vessel walls. α_4_β_1_ and β_2_ integrins, expressed on neutrophil cell membrane, are required and strongly mediate rolling and firm adhesion, crawling and transmigration steps of adhesion cascade [14, 41] as their activation is an essential step of this complex process. To understand whether HBOT could influence integrin-mediated neutrophil adhesion, we performed cell adhesion assays to fibronectin (FN) or fibrinogen (Fg), ligands for α_4_β_1_ and β_2_ integrins respectively, on neutrophils isolated from patients with chronic non-healing wound, receiving standard wound care alone (control group) or undergoing HBOT (HBOT group). Adhesion of neutrophils obtained from patients belonging to both control and HBOT groups was significantly reduced by the addition of integrin specific antibodies able to block integrin functions (Fig 2), demonstrating that neutrophil adhesion to fibronectin or fibrinogen was mainly mediated by α_4_β_1_ or β_2_ integrin, respectively. Moreover, as shown in Fig 2, exposure to HBO induces a significant reduction of neutrophil adhesion mediated by either β_2_ or α_4_β_1_ integrins (Fig 2, panel a and b, respectively). Interestingly, this reduction of neutrophil adhesive properties is retained throughout the duration of HBO treatment and up to one month after the last HBOT session.

In addition, these data showed that adhesive properties of α_4_β_1_ integrin expressed on neutrophils were impaired during HBOT although its expression was not modified both at mRNA and protein levels (as shown in Fig 1). To better understand the involvement of α_4_β_1_ integrin in neutrophil adhesion, we used a conformation-specific antibody that recognizes a specific epitope on integrin β_1_ subunit exposed only in a defined structural conformation [42]. Integrins exist in three major conformations: a bent or inactive, an intermediate-active and an open high-activity conformation [43]. To monitor conformational changes in integrin subunits it is therefore possible to use conformation-specific antibodies [44]. We employed the PE-conjugated HUTS-21 mAb to determine whether the reduced adhesive properties of neutrophils mediated by α_4_β_1_ integrin during HBOT are due to a conformational change of β_1_ subunit, indicative of its activation status. HUTS-21 antibody recognizes a ligand-induced binding site that is hidden in the inactive conformation, but it is exposed when the agonist binds or upon partial integrin activation, namely when the integrin is in a high affinity conformation. The epitope recognized by HUTS-21 antibody is located in the hybrid domain of β_1_ integrin subunit [42]. PE-conjugated anti-β_1_ HUTS-21 mAb was added to neutrophils in the presence of fibronectin (10 μg/mL), and fluorescence was measured by flow cytometry. Exposure to HBOT induced a significant reduction of HUTS-21 mAb binding to neutrophils, throughout the duration of the treatment and up to one month after the last HBOT session (Fig 2, panel c), meaning that α_4_β_1_ integrin expressed on neutrophil surface was mainly present in a low affinity conformation. Neutrophil deriving from control patient group did not show any variation in α_4_β_1_ integrin conformation (Fig 2, panel c). These data demonstrated that HBOT significantly reduced α_4_β_1_ integrin-mediated adhesive properties of neutrophils, not reducing its expression but rather changing its shape towards a lower activity conformation.

### Evaluation of ulcer size and inflammatory cytokines in neutrophils and in plasma after HBOT

**Fig 3:**
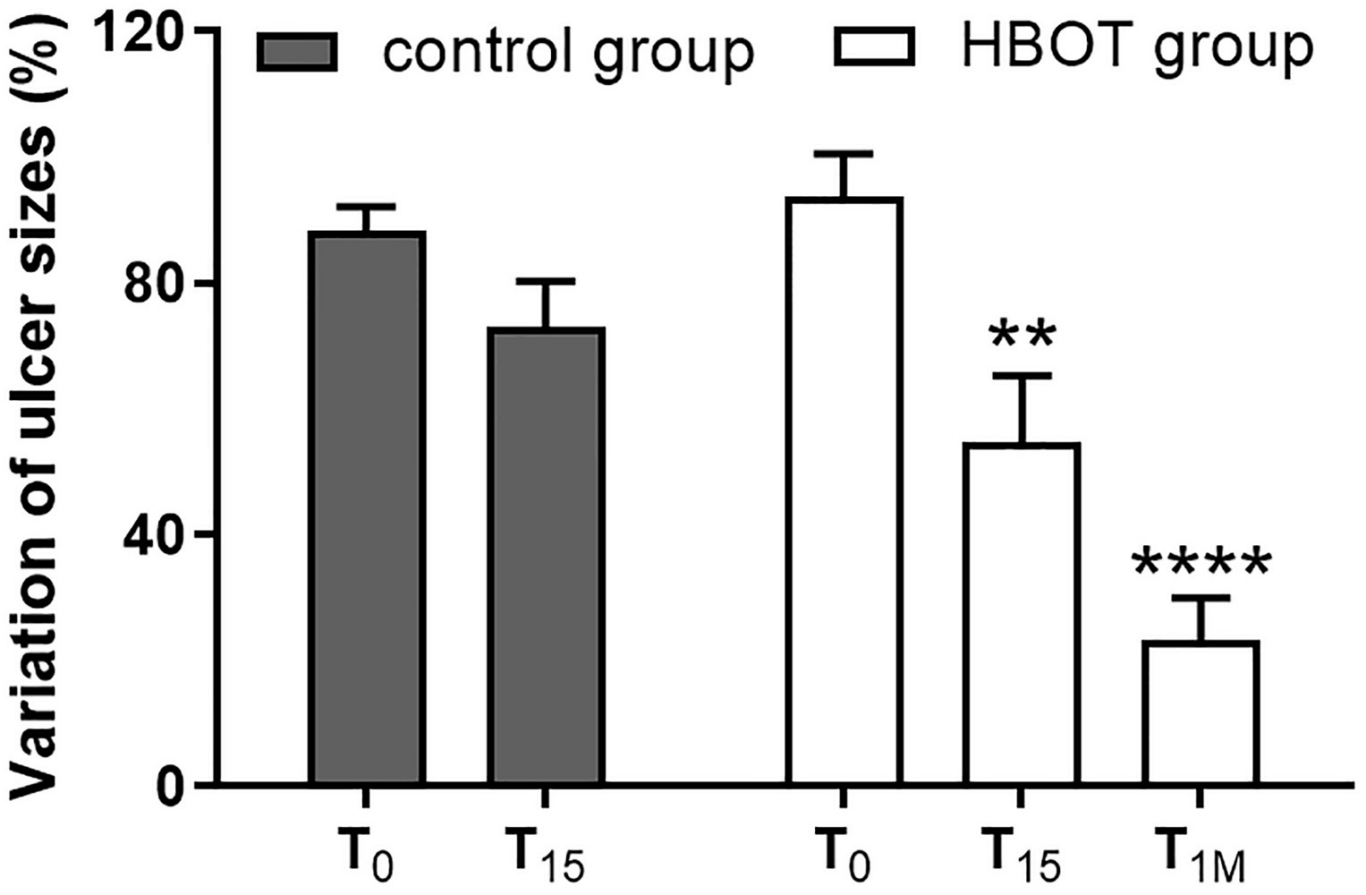
Variation of ulcer sizes at different time points during and after HBO therapy in patients with chronic non-healing wounds. Ulcer sizes were determined for control group patients during the first evaluation (T_0_) and after fifteen days of conventional wound therapy (T_15_); for patients in HBOT group before (T_0_), after 15 HBO sessions (T_15_) and one month after the last HBO treatment (T_1M_). Data, expressed as variation of the percentage in ulcer sizes and related to the basal value (T_0_), represent the mean ± standard deviation of individual samples (control group n = 15; HBOT group n = 15). **p<0.01, **** p < 0.0001 versus T_0_, both control and HBOT group.

**Fig 4:**
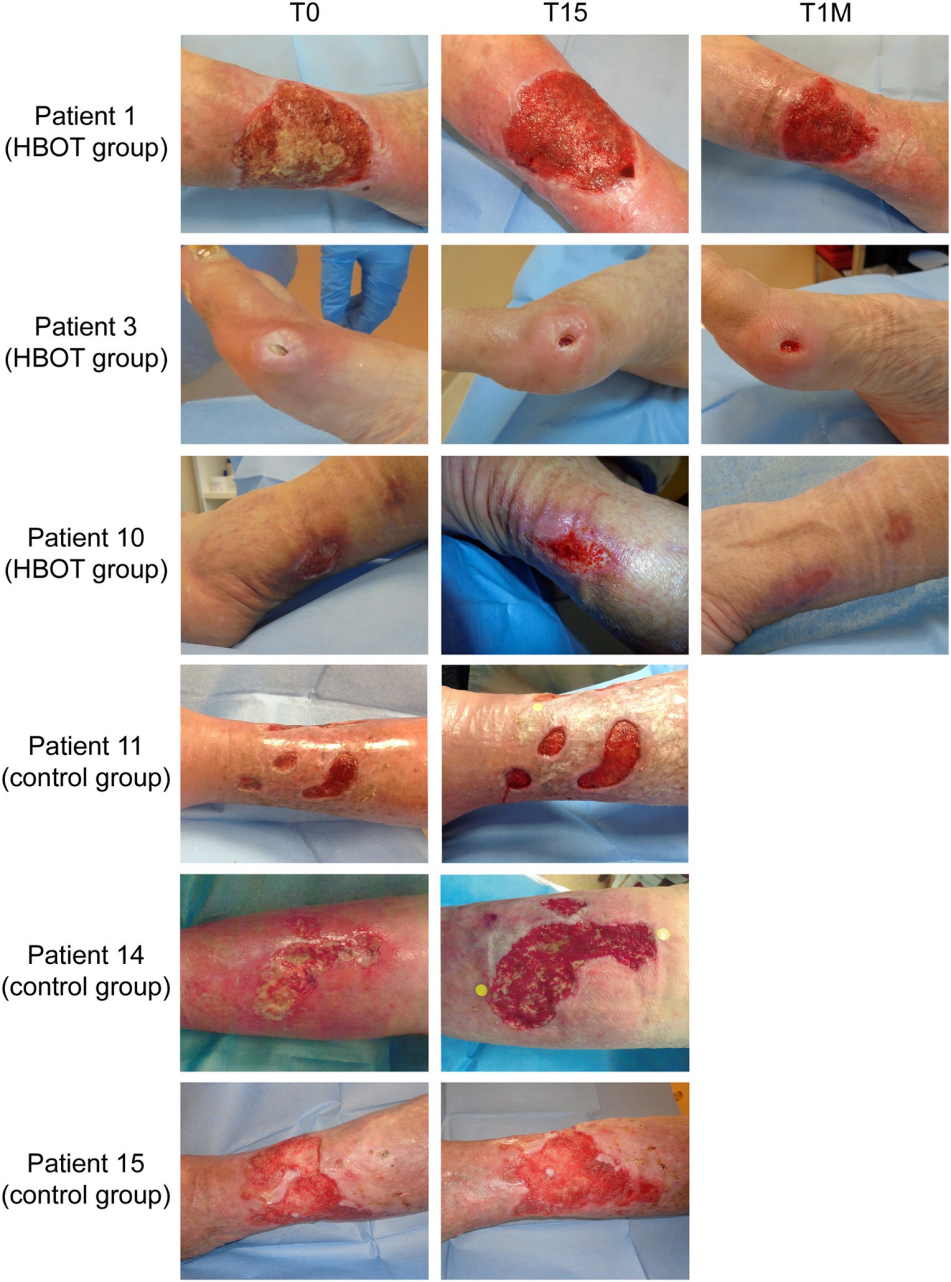
Wound healing progression during and after HBO therapy in patients with chronic non-healing wounds, shown qualitatively. Images of the same ulcer were acquired before (T_0_), after 15 HBO sessions (T_15_) and one month after the last HBO treatment (T_1M_) for patients of HBOT group; during the first evaluation (T_0_) and after fifteen days of standard wound therapy (T_15_) for control group patients. Three representative sets of wound images for each group are shown.

To compare the trend of the studied parameters with the progress of the inflammation, ulcer sizes were measured for control group patients during the first evaluation and after fifteen days of conventional wound therapy. For patients in HBOT group ulcer sizes were evaluated before starting HBOT, after 15 sessions at 245 kPa (FiO_2_ in mask 100%) per 90 minutes in cycles for 20 minutes separated by 3-minute medical air breathing intervals, and one month after ending HBOT. Several studies have proposed HBOT as noninvasive adjunctive therapy that may contribute to heal chronic wounds [45]. We opted for Falanga wound bed preparation score [31] as monitoring methodology to follow ulcers evolution. After 15 treatments (three weeks; five exposures/week) with HBOT, we observed a significant reduction of ulcer size (Fig 3) and the mean wound areas were decreased about 60% in comparison to basal wound area. An 80% decrement compared to basal area value at T_0_ was observed one month after the last HBO treatment (Fig 3) and an improvement in wound score was observed after 15 sessions of HBOT. In addition, this progressive wound closure was maintained one month after the last HBOT sessions (Table 3), leading at least in four patients to complete healing of the ulcer. In control group patients we observed only a slight, although not significant, improvement in ulcer sizes (Fig 3) and in Falanga score (Table 4) after 15 days of standard wound care alone. Representative images of wounds captured for HBOT group patients prior to exposure to HBOT, after the fifteenth session and one month after the last HBO treatment and for patients belonging to control group during the first evaluation and after fifteen days of standard wound therapy are shown in Fig 4.

**Table 3:** Variation of Falanga wound bed preparation score of HBOT group of patients. Letters refer to wound appearance and numbers to wound exudate score as described in methods (see Table 2).

	Falanga score		
Patient n.	T_0_	T_15_	T_1M_
**1**	C3	B2	A1
**2**	D1	B2	A1
**3**	C2	B2	A1
**4**	C2	B1	B1
**5**	C3	B1	A1
**6**	C3	B2	A1
**7**	C1	A1	healed
**8**	C2	B2	A1
**9**	C2	A2	healed
**10**	B1	A1	Healed
**11**	C2	C1	B1
**12**	C1	B2	A1
**13**	C3	B2	A1
**14**	C3	B1	Healed
**15**	C2	B1	A1

**Table 4:** Variation of Falanga wound bed preparation score of control group of patients. Letters refer to wound appearance and numbers to wound exudate score as described in methods (see Table 2).

	Falanga score	
Patient n.	T_0_	T_15_
**1**	C1	A1
**2**	B2	A1
**3**	B2	Healed
**4**	C2	C1
**5**	B1	B1
**6**	B2	B2
**7**	B2	A1
**8**	B2	B1
**9**	B2	B2
**10**	B1	B1
**11**	C2	B2
**12**	C1	B2
**13**	B2	B1
**14**	C3	B2
**15**	B2	B2

**Fig 5:**
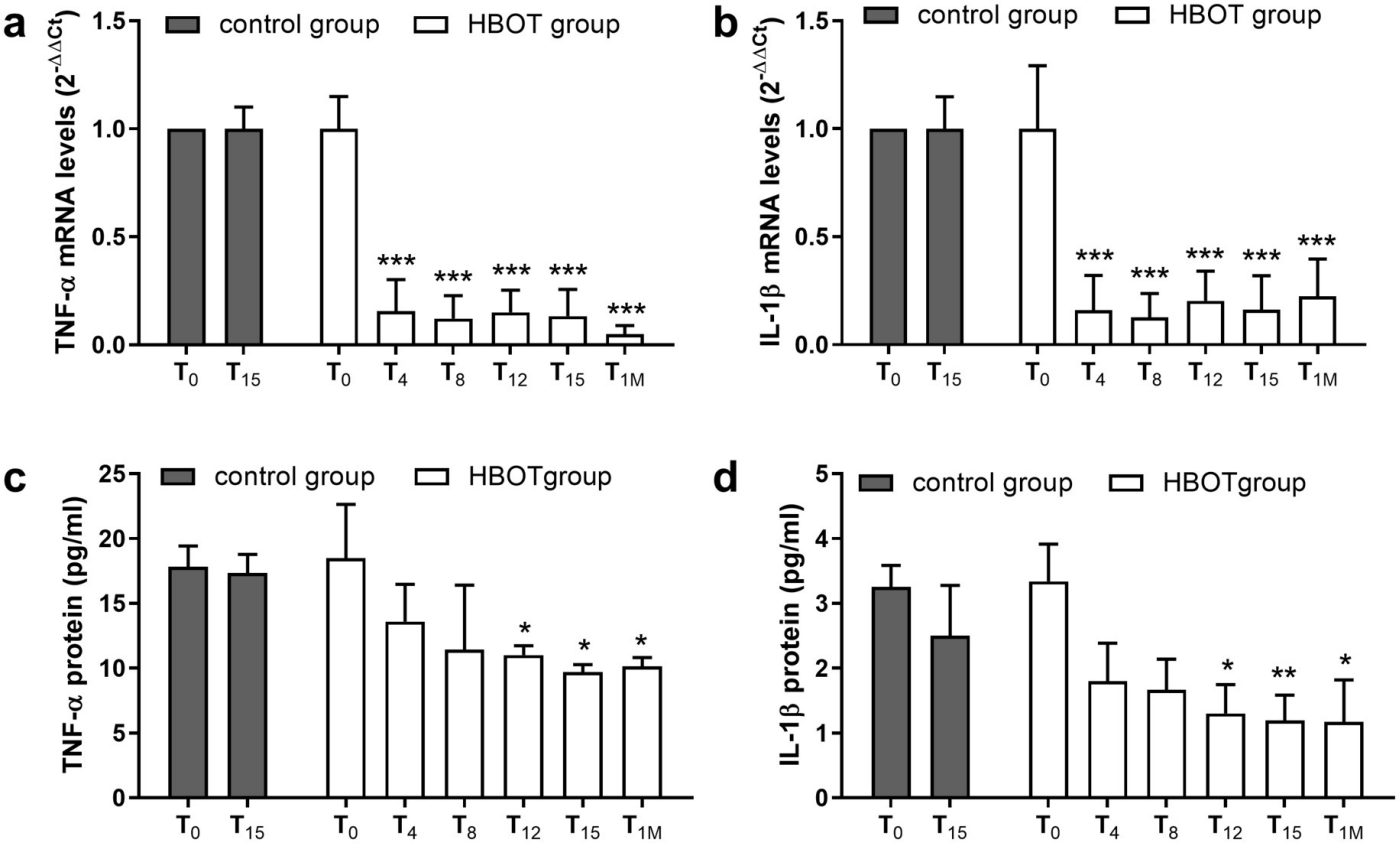
Effects of HBOT on TNF-α and IL-1β evaluated as mRNA levels in human primary neutrophils and circulating protein levels in plasma. HBO treatment significantly reduced TNF-α (panel a) and IL-1β (panel b) mRNA levels in neutrophils isolated from blood samples of patients with chronic non-healing wounds undergoing HBOT or from control group. Moreover, HBOT induced a significant reduction in circulating inflammatory cytokines TNF-α (panel c) and IL-1β (panel d) starting from the twelfth HBO session up to one month after the last HBO treatment in HBOT group patients. No changes in pro-inflammatory cytokine levels, both at neutrophil mRNA and circulating protein levels, were observed in control patients. Data are expressed as mean ± standard deviation of individual samples, carried out in triplicate (control group n = 15; HBOT group n = 15). * p < 0.05; ** p < 0.01; *** p < 0.001 versus T_0_, both control and HBOT group.

To monitor the neutrophilic inflammation in HBOT, we evaluated mRNA levels of the pro-inflammatory cytokines TNF-α and IL-1β in neutrophils. As shown in Fig 5 (panel a and b), HBOT induces a significant reduction of both TNF-α and IL-1β mRNA levels soon after the fourth session, which is maintained up to one month from the last HBO session. These results suggest a reduction in the inflammatory state induced by HBOT. To confirm this hypothesis, we measured circulating levels of inflammatory markers TNF-α and IL-1β using a commercial ELISA assay. We observed a significant reduction of TNF-α and IL-1β circulating levels after the twelfth session of HBO in patients belonging to HBOT group (Fig 5, panel c and d); the reduced levels of pro-inflammatory cytokines were maintained until one month after the last HBO session. In control group patients the levels of circulating TNF-α and IL-1β did not vary after 15 days of standard wound care (Fig 5), indicating a sustained inflammatory status.

In a previous paper [29], we developed a novel class of dehydro-β-proline-containing peptidomimetics, designed to be effective as α_4_β_1_ integrin ligands, on the basis of the fundamental requirements for integrin interactions with bioactive ligands. In general, features for effective ligand-receptor interaction are the presence of a carboxylate group, a donor of H-bond as an amide moiety in the central part of the molecule and a lipophilic chain mimicking the leucine side chain present in the VCAM-1 recognition sequence. Moreover, the presence of 4([(N-2-methylphenyl)ureido]-phenylacetyl motif (PUPA) greatly enhances bioactivity, as observed for the very effective ligand α_4_β_1_ BIO1211 [29]. Conformational studies suggested an almost linear disposition of the molecule, as could be expected on the basis of structural restraints. This is in agreement with the typically preferred conformation reported for other active α_4_β_1_-integrin ligands. The synthesized products showed to be effective and selective as α_4_β_1_ integrin antagonists and display IC_50_ values in the nanomolar range in cell adhesion assays. Among them, RG66 possesses a significant affinity and selectivity for integrin α_4_β_1_ in inhibiting cell adhesion to VCAM-1 (IC_50_ 10 ± 3 nM) performed on T lymphocyte cells (Jurkat cell line) [29].

Targeting leukocyte integrins (such as α_4_β_1_) has proven applications in several diseases such as Crohn’s disease, ulcerative colitis and multiple sclerosis [46, 47]. The rationale for targeting this class of integrins is to modulate aberrant immune cell migration and adhesion during the inflammatory processes. For these reasons, antagonists specifically designed to target this integrin are actively searched for clinical applications [48].

**Fig 6:**
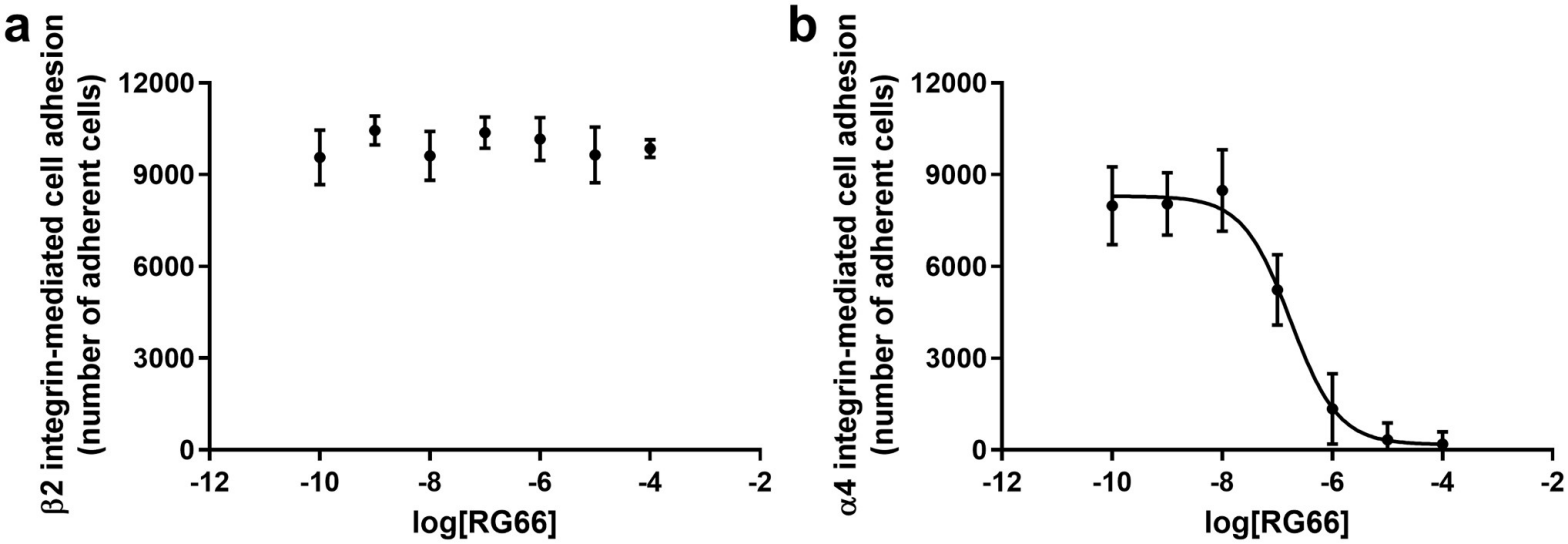
Effects of integrin antagonist RG66 on integrin-mediated neutrophil adhesion. Integrin antagonist RG66 does not influence neutrophil adhesion mediated by β_2_ integrins (panel a); on the contrary, RG66 reduces α_4_β_1_ integrin-mediated neutrophil adhesion to fibronectin (FN) in a concentration-dependent manner (IC_50_ 0.17 ± 0.03 μM) (panel b). The effects of RG66 (10^−4^–10^−10^ M) on neutrophil adhesion mediated by β_2_ (panel a) or α_4_ (panel b) integrins were evaluated by adhesion assay to Fg or FN respectively, as described in method section. Neutrophils were isolated from blood samples of patients (with chronic non-healing wounds) undergoing HBOT. A representative concentration-response curve at T_8_ is reported. Data are expressed as mean ± standard deviation, carried out in triplicate (HBOT group, n = 15).

In order to understand if RG66 could be useful to reduce neutrophil migration and adhesion in inflammatory diseases, neutrophils obtained from patients undergoing HBOT or receiving conventional wound therapy alone were used for cell adhesion assay with different concentrations (10^−4^–10^−10^ M) of our compound. RG66 was able to reduce neutrophil cell adhesion mediated by α_4_β_1_ integrin in a concentration-dependent manner with IC_50_ value in the submicromolar range (α_4_β_1_ vs FN, IC_50_ 0.17 ± 0.03 μM) (Fig 6 and S2 Fig). Furthermore, its antagonistic effect was maintained on neutrophils deriving from patients undergoing HBOT at various time points considered (T_0_, T_4_, T_8_, T_12_, T_15_ and T_1M_) and even on neutrophils obtained from control group patients (Fig 6 and S2 Fig). On the contrary, confirming its specificity towards α_4_β_1_ integrin, ligand RG66 did not modify neutrophil adhesion mediated by α_L_β_2_ integrin (Fig 6 and S3 Fig). This was further verified by the ability of an anti-β_2_ integrin antibody to significantly reduce neutrophil cell adhesion to fibrinogen even in presence of RG66 (0.1 μM) (S3 Fig, panel c).

In a previous study [49], we have already demonstrated that blocking α_4_β_1_ integrin with a specific antibody or with the antiallergic drug levocabastine, able to bind to α_4_β_1_, was an interesting strategy to reduce immune cell adhesion to endothelial cells (HUVEC cell line) and to reduce α_4_β_1_-mediated eosinophil recruitment in an animal model of allergic conjunctivitis. Further studies are needed to better deepen the activity of integrin antagonists, such as RG66, on neutrophil adhesion to endothelial cells, on neutrophil recruitment at wound site and the role played by HBOT.

## Discussion

HBOT is used as a safe adjunctive therapy for chronic non-healing wound [45, 50–53]. Wound healing is a complex and a tightly regulated process that requires a well-orchestrated interplay of molecular and cellular events and that can be divided into several stages: hemostasis, inflammation, re-epithelialization and tissue remodeling [54]. Among different cells activated by immune response, neutrophils are among the first cells recruited to the wound site through an integrin-mediated recruitment process and they play a key role in wound healing through the release of reactive oxygen species (ROS) and proteolytic enzymes. In addition, neutrophils may contribute to prolong inflammation and to induce tissue damage; these effects prevent proper wound healing and lead to chronic wound [12].

In the present study, we have investigated the effects of HBO on α_4_ and β_2_ integrins expression and functions in neutrophils obtained from patients with chronic non healing ulcers undergoing HBOT or standard wound therapy alone. We observed that treatment with HBO significantly reduces β_2_ integrin expression on neutrophils, while α_4_ integrin levels remain unchanged. Additionally, β_2_ integrin decrement is maintained at various time points during HBOT and up to one month after the last HBO session.

In cell adhesion assays employing neutrophils obtained from patients with chronic wounds undergoing HBOT, treatment with HBO induces a significant reduction of integrin-mediated adhesive properties of neutrophils through the engagement of both α_4_ and β_2_ integrins. HBO did not affect α_4_ integrin expression, but it reduces α_4_-mediated neutrophil adhesion probably inducing a switch towards the low affinity, inactive conformation, as demonstrated by using HUTS-21 conformational sensitive antibody.

In neutrophils obtained from control group patients, we did not observe any change in α_4_ and β_2_ integrin expression and in integrin-mediated adhesive properties after 15 days of standard wound care.

Previous studies have shown that HBO diminishes β_2_ integrin-mediated neutrophil adhesion but it does not alter surface expression of β_2_ integrins on neutrophils [24, 25, 28, 55, 56]. Thom et al. [28] have observed that *in vitro* exposure of polymorphonuclear leukocytes (PMN) to HBO inhibits their binding to fibrinogen-coated surfaces in a dose-dependent way without affecting membrane expression of β_2_ integrins on resting or activated PMN, suggesting that HBO induces an alteration of β_2_ integrin activity, probably linked to impaired synthesis of 8-bromoguanosine 3’,5’-cyclic monophosphate (cGMP). Kalns et al. [56] have observed that in neutrophils obtained from healthy volunteers, one session of HBO specifically blocks α_M_β_2_ integrin-mediated functions at 2 and 6 hours after the exposure. Moreover, although the effect of HBO treatment on neutrophil adhesion is not strictly limited to the time of hyperbaric session, it is transient and no longer significant 24 h after HBO treatment [56]. In an *in vitro* model [24] developed to mimic the events occurring in microcirculation during ischemia reperfusion (IR) injury, it has been observed that HBO treatment inhibits neutrophil adhesion to ICAM-1 and decreases β_2_ integrin polarization induced by IR; two early and key steps in the inflammatory cascade of IR injury, that mediate, respectively, neutrophil recruitment and firm adhesion to endothelial cells. A more recent study [25] confirmed that HBO reduced neutrophil adhesion to endothelial cells *in vitro*, without altering α_M_ and β_2_ integrins, L-selectin and Platelet endothelial cell adhesion molecule (PECAM-1) expression on neutrophil cell membranes but inducing an impairment in integrin functions mediated by S-nitrosation of actin. In fact, this posttranslational modification of cytoskeletal actin alters its polymerization and as a consequence the formation of actin-integrin complexes [25] and contributes to the redistribution of integrins on neutrophil surface that ultimately influences integrin-mediated cell adhesion. The reduced adhesive properties of neutrophils may also be partially due to the decrease in both ICAM-1 and VCAM-1 expression on endothelial cells [25]. In fact, it has also been confirmed that HBO may have a synergistic inhibitory effect influencing the functions of both neutrophils and vascular endothelium [57], in the latter reducing ICAM-1 expression.

To our knowledge, this is the first study in which integrin expression and functions have been evaluated in human primary neutrophils obtained from patients with chronic wounds and throughout a prolonged HBOT and compared to those isolated from patients undergoing standard wound therapy alone. In contrast to the *in vitro* studies previously reported, we observed a significant reduction of neutrophil β_2_ integrin expression; hence the data cannot be compared as these studies were conducted *in vitro* on neutrophils obtained from healthy volunteers.

The patients enrolled in this study display a chronic non-healing wound condition that determines an increased inflammatory state [58] strongly reduced by HBOT. In fact, we observed a significant decrement of mRNA levels of pro-inflammatory cytokines TNF-α and IL-1β in neutrophils after HBO exposure, an effect that is maintained up to one month after ending HBOT. Moreover, circulating levels of TNF-α and IL-1β, evaluated in plasma, were significantly reduced by HBOT if compared to pro-inflammatory cytokine levels measured in control patients. TNF-α and IL-1β are pro-inflammatory cytokines that act as key mediators of the inflammatory process and are overexpressed in the inflammatory phase of the wound healing process [59]. Low levels of both mRNAs may be related to a reduction of the corresponding cytokine that may induce wound healing while, on the contrary, high levels as those found in chronic wound, can counteract healing with detrimental consequences [60]. Therefore, among other effects, HBO may contribute to inflammation treatment by reducing some specific pro-inflammatory mediators [58] and by diminishing leukocyte recruitment and their detrimental effects at chronic wound through the reduction of integrins expression and functions. Moreover, in parallel to decreased expression of pro-inflammatory cytokines, we observed a significant reduction of ulcer area, leading in four patients to complete wound healing. These data further are in agreement with a role of HBOT in the acceleration of chronic wound healing, as previously described [45, 61].

The efficacy of HBOT appears to derive from a complex combination of overlapping systemic events and local alterations within the wound. The sustained effects of HBOT observed in this study could be due to the overall reduction of the inflammatory state of the patients, as confirmed by the reduction of both circulating inflammatory cytokines and wound area. Moreover, previous studies have demonstrated that HBO increases nitric oxide levels in the bone marrow thereby increasing the release of endothelial progenitor cells into the circulation. The mobilization of endothelial progenitors contributes to angiogenesis and wound healing [62, 63]. In addition, an increment of circulating CD34^+^ cells after 20 HBO sessions was observed, although the overall circulating white cells were not significantly increased [64]. Further studies would be necessary to unravel the effects of HBOT on bone marrow functions.

To further enforce the blockade of α_4_β_1_ integrin, we propose to investigate in future studies the possibility to combine HBO treatment with an integrin antagonist as RG66, that is a selective α_4_ integrin antagonist [29], able to strongly reduce α_4_β_1_-mediated adhesion of neutrophils deriving from patients with chronic wound. Therefore, RG66 has proven to be effective in further reducing integrin-mediated adhesion also in primary neutrophils already exposed to HBO. Leukocyte integrins are considered as interesting therapeutic targets for the development of new drugs useful to treat inflammatory diseases. In fact, several drugs targeting integrins such as α_4_ have proven to be effective for the therapy of Crohn’s disease, ulcerative colitis and multiple sclerosis [46, 47, 65, 66] and others are in development to treat ocular diseases [44] or to reduce scar formation [67]. Therefore, further studies on integrin antagonists are necessary to better understand the possibility to develop a combined therapy with HBOT for the treatment of chronic wounds.

In conclusion, we demonstrate that HBOT promotes wound healing and a reduction of inflammatory cytokines in patients with chronic non-healing wound. The cell adhesion function of both neutrophilic integrins α_4_β_1_ and β_2_ is significantly reduced as well as expression of β_2_ integrins. We propose this study as a starting point to better evaluate, in the future, the possibility to use a combined therapy between HBOT and integrin antagonists to strongly reduce neutrophils recruitment and lead to a better and faster wound healing.

## Supporting information

S1 Fig Reactions and conditions for the synthesis of RG66. (a) methyl malonyl chloride, TEA, DCM, r.t, 3h, yield 90–95%. (b) Grubbs-Hoveyda II catalyst (3% mol), MTBE, reflux, 3h yield 80–95%. (c) TFA, DCM, r.t. 12h, yield >90% (d) HBTU, DIPEA, DCM, 4-(aminomethyl)aniline, r.t, overnight, yield 75–85%. (e) 2-methylbenzene isocyanate, DCM, r.t, 6h, yield 80–88%. (f) K2CO3, THF/H2O, r.t. 2h, yield >90%.
(TIFF)

S2 Fig Effects of integrin antagonist RG66 on α_4_β_1_ integrin-mediated neutrophil adhesion. a) Integrin antagonist RG66 reduces α_4_β_1_ integrin-mediated neutrophil adhesion to fibronectin (FN) in a concentration-dependent manner at various time point considered (T_0_, T_4_, T_8_, T_12_, T_15_ and T_1M_) for HBOT group patients. The effects of RG66 on neutrophil adhesion mediated by α_4_β_1_ integrin were evaluated by adhesion assay to FN, as described in method section. Neutrophils were isolated from blood samples of patients (with chronic non-healing wounds) undergoing HBOT, obtained before (T_0_) and immediately after the fourth (T_4_), the eighth (T_8_), the twelfth (T_12_), the fifteenth (T_15_) HBOT sessions and one month after the last HBO treatment (T_1M_) and b) for patients belonging to control group during the first evaluation (T_0_) and after fifteen days of standard wound therapy (T_15_). Data are expressed as mean ± standard deviation of individual samples, carried out in triplicate (control group n = 15; HBOT group n = 15).
(TIF)

S3 Fig Effects of integrin antagonist RG66 on β_2_ integrin-mediated neutrophil adhesion. a) Integrin antagonist RG66 does not modify β_2_ integrin-mediated neutrophil adhesion to fibrinogen (Fg) at various time point considered (T_0_, T_4_, T_8_, T_12_, T_15_ and T_1M_) for HBOT group patients. The effects of RG66 on neutrophil adhesion mediated by β_2_ integrin were evaluated by adhesion assay to Fg, as described in method section. Neutrophils were isolated from blood samples of patients (with chronic non-healing wounds) undergoing HBOT, obtained before (T_0_) and immediately after the fourth (T_4_), the eighth (T_8_), the twelfth (T_12_), the fifteenth (T_15_) HBOT sessions and one month after the last HBO treatment (T_1M_) and b) for patients belonging to control group during the first evaluation (T_0_) and after fifteen days of standard wound therapy (T_15_). c) Adhesion to Fg, mediated by β_2_ integrins, is significantly prevented in neutrophils treated with a monoclonal antibody anti-β_2_ even in presence of RG66 (0.1 μM). Data are expressed as mean ± standard deviation of individual samples, carried out in triplicate (control group n = 15; HBOT group n = 15). ** p < 0.01 versus T_0_.
(TIF)

S4 Fig HBOT effects on α_4_ (panels a and c) and β_2_ integrin (panels b and d) protein levels in neutrophils deriving from patients receiving standard wound care alone (control group) and patients undergoing HBOT for 15 sessions (HBOT group). Data from individual representative patients (as in Fig 4) during HBOT are shown. The effects of HBOT on integrin expression were evaluated by flow cytometry (measuring integrin expressed on cell surface). Data are expressed as mean fluorescence intensity (MFI) ± standard deviation carried out in triplicate at each time point (control group n = 15; HBOT group n = 15). MFI values for respective isotype control monoclonal antibody were set to 0.
(TIF)
