# Supplementary figures and images for: Integrin-mediated adhesive properties of neutrophils are reduced by hyperbaric oxygen therapy in patients with chronic non-healing wound
Source: PLoS One. 2020 Aug 18;15(8):e0237746. doi: 10.1371/journal.pone.0237746 (PMC7433869; doi:10.1371/journal.pone.0237746)

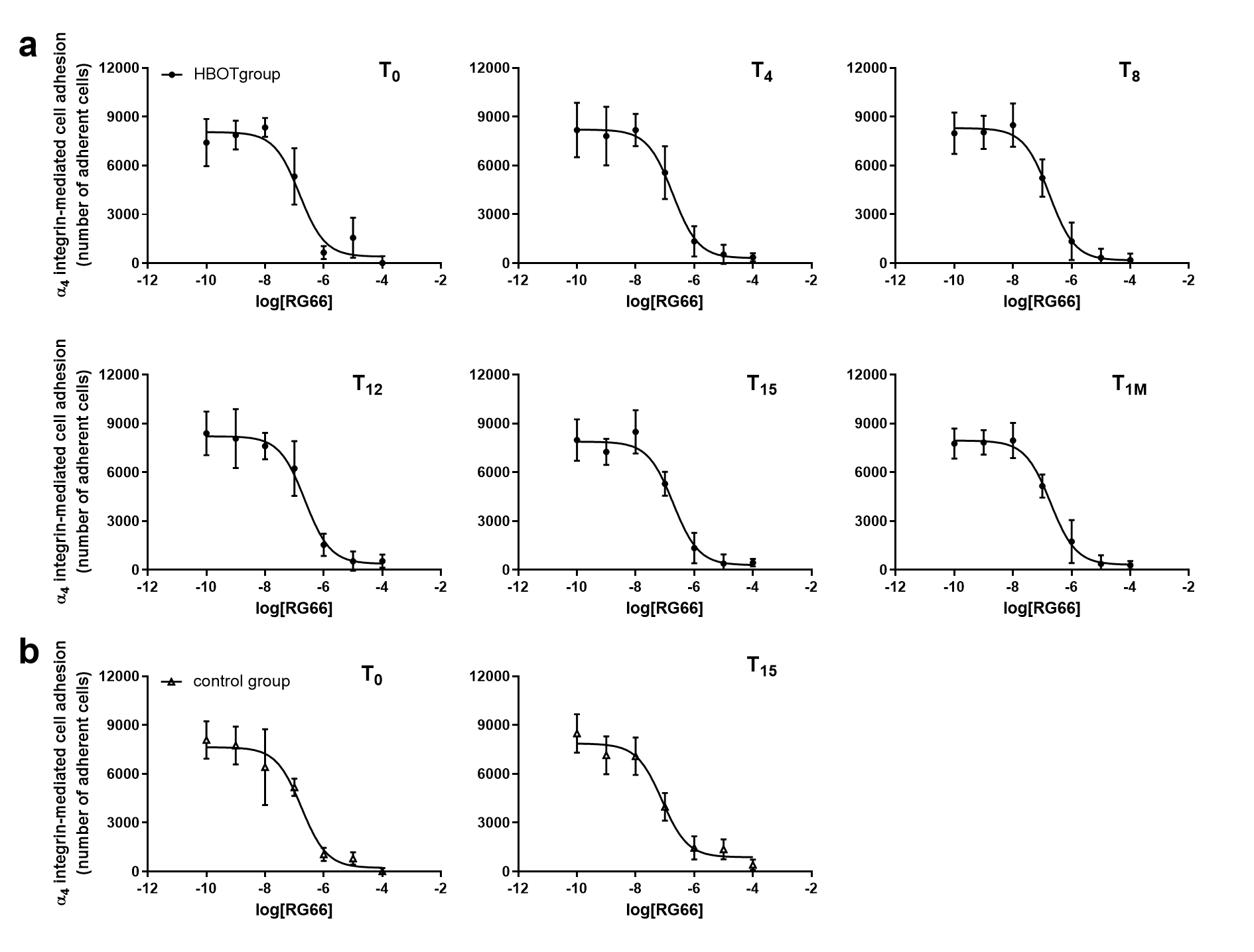

Supplement: S2 Fig — a) Integrin antagonist RG66 reduces α4β1 integrin-mediated neutrophil adhesion to fibronectin (FN) in a concentration-dependent manner at various time point considered (T0, T4, T8, T12, T15 and T1M) for HBOT group patients. The effects of RG66 on neutrophil adhesion mediated by α4β1 integrin were evaluated by adhesion assay to FN, as described in method section. Neutrophils were isolated from blood samples of patients (with chronic non-healing wounds) undergoing HBOT, obtained before (T0) and immediately after the fourth (T4), the eighth (T8), the twelfth (T12), the fifteenth (T15) HBOT sessions and one month after the last HBO treatment (T1M) and b) for patients belonging to control group during the first evaluation (T0) and after fifteen days of standard wound therapy (T15). Data are expressed as mean ± standard deviation of individual samples, carried out in triplicate (control group n = 15; HBOT group n = 15). (TIF) [file pone.0237746.s002.tif]

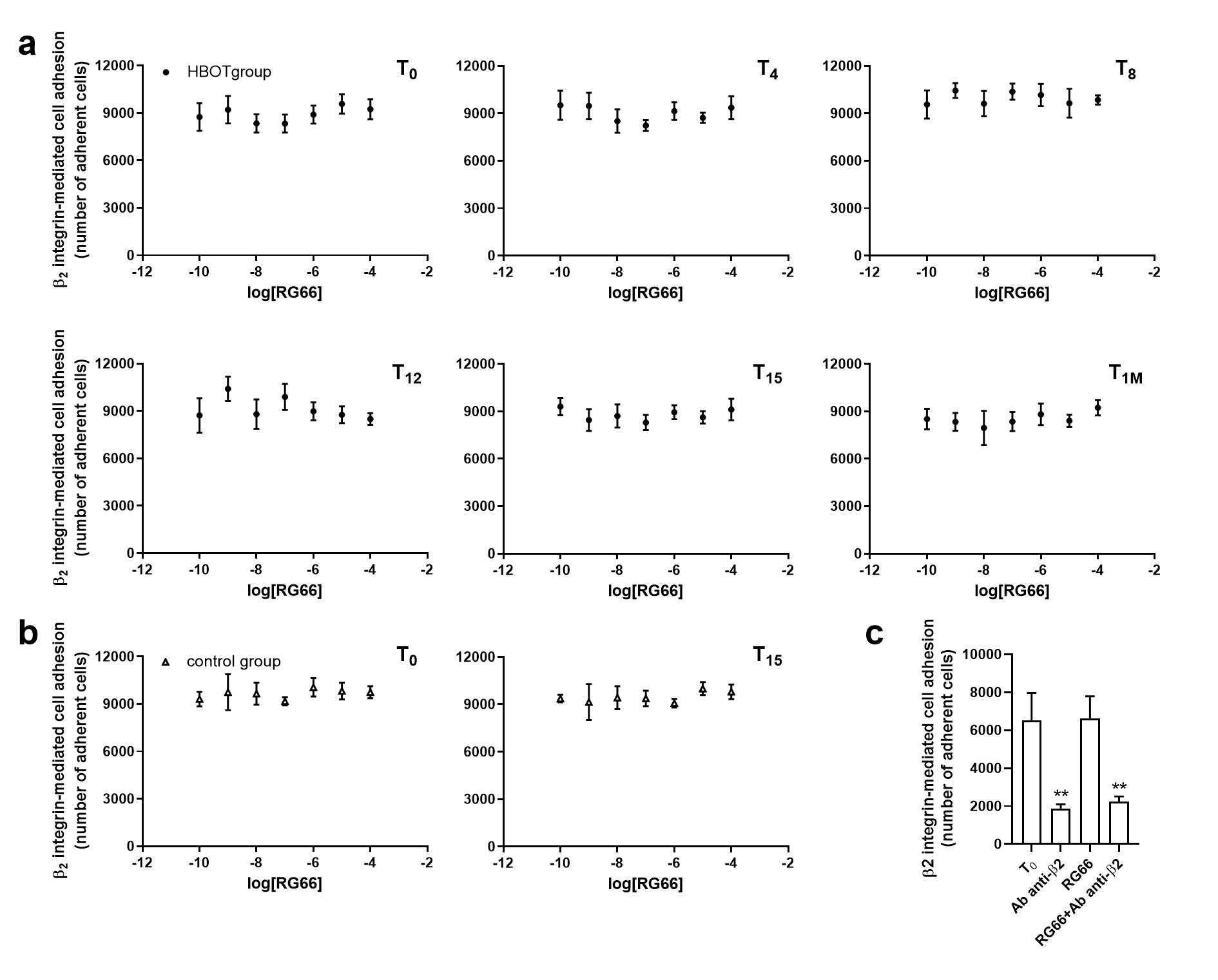

Supplement: S3 Fig — a) Integrin antagonist RG66 does not modify β2 integrin-mediated neutrophil adhesion to fibrinogen (Fg) at various time point considered (T0, T4, T8, T12, T15 and T1M) for HBOT group patients. The effects of RG66 on neutrophil adhesion mediated by β2 integrin were evaluated by adhesion assay to Fg, as described in method section. Neutrophils were isolated from blood samples of patients (with chronic non-healing wounds) undergoing HBOT, obtained before (T0) and immediately after the fourth (T4), the eighth (T8), the twelfth (T12), the fifteenth (T15) HBOT sessions and one month after the last HBO treatment (T1M) and b) for patients belonging to control group during the first evaluation (T0) and after fifteen days of standard wound therapy (T15). c) Adhesion to Fg, mediated by β2 integrins, is significantly prevented in neutrophils treated with a monoclonal antibody anti-β2 even in presence of RG66 (0.1 μM). Data are expressed as mean ± standard deviation of individual samples, carried out in triplicate (control group n = 15; HBOT group n = 15). ** p < 0.01 versus T0. (TIF) [file pone.0237746.s003.tif]

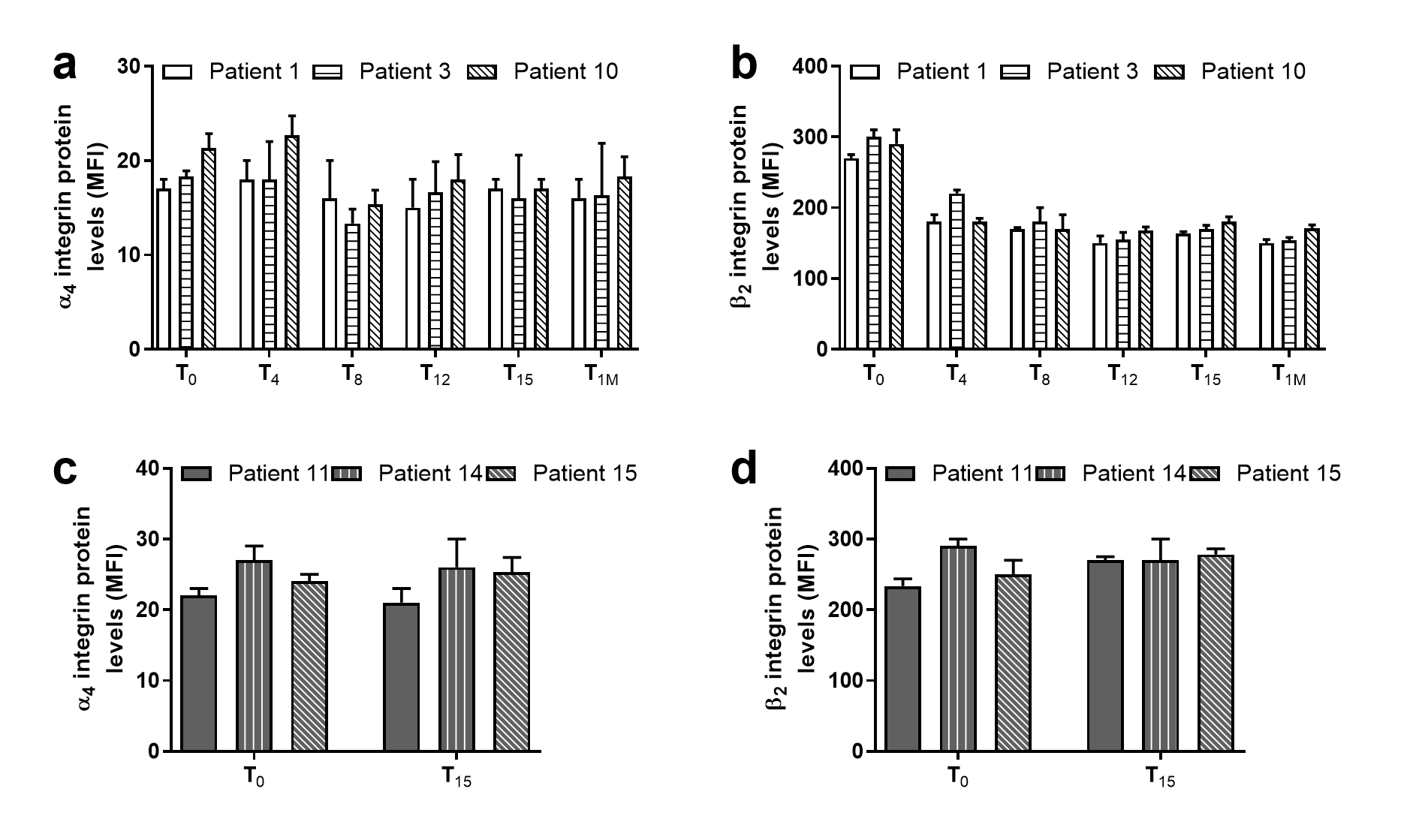

Supplement: S4 Fig — Data from individual representative patients (as in Fig 4) during HBOT are shown. The effects of HBOT on integrin expression were evaluated by flow cytometry (measuring integrin expressed on cell surface). Data are expressed as mean fluorescence intensity (MFI) ± standard deviation carried out in triplicate at each time point (control group n = 15; HBOT group n = 15). MFI values for respective isotype control monoclonal antibody were set to 0. (TIF) [file pone.0237746.s004.tif]
